# Supplementary material for: SOX11 regulates SWI/SNF complex components as member of the adrenergic neuroblastoma core regulatory circuitry
Source: Nat Commun. 2023 Mar 7;14:1267. doi: 10.1038/s41467-023-36735-2 (PMC9992472; doi:10.1038/s41467-023-36735-2)
Supplement: Supplementary file 1 — Supplementary Information [file 41467_2023_36735_MOESM1_ESM.pdf]

Supplementary Information

SOX11 regulates SWI/SNF complex components as member of the adrenergic neuroblastoma core regulatory circuitry

Decaesteker, Louwagie et al.

Supplementary Figure 1: Rare focal amplifications and lineage-specific expression of *SOX11* in neuroblastoma (1 of 2)

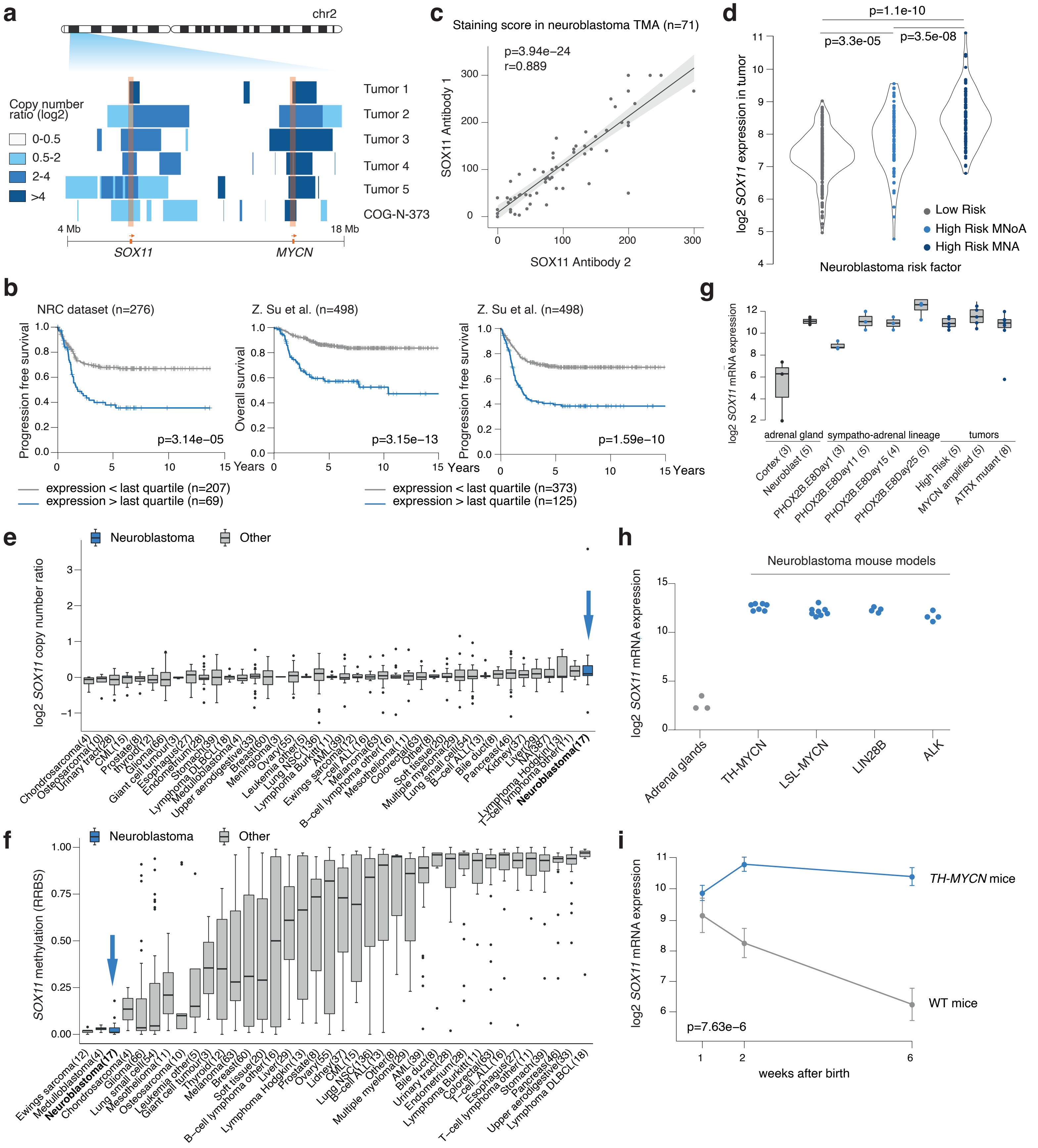

Supplementary Figure 1: Rare focal amplifications and lineage-specific expression of *SOX11* in neuroblastoma (2 of 2)

j

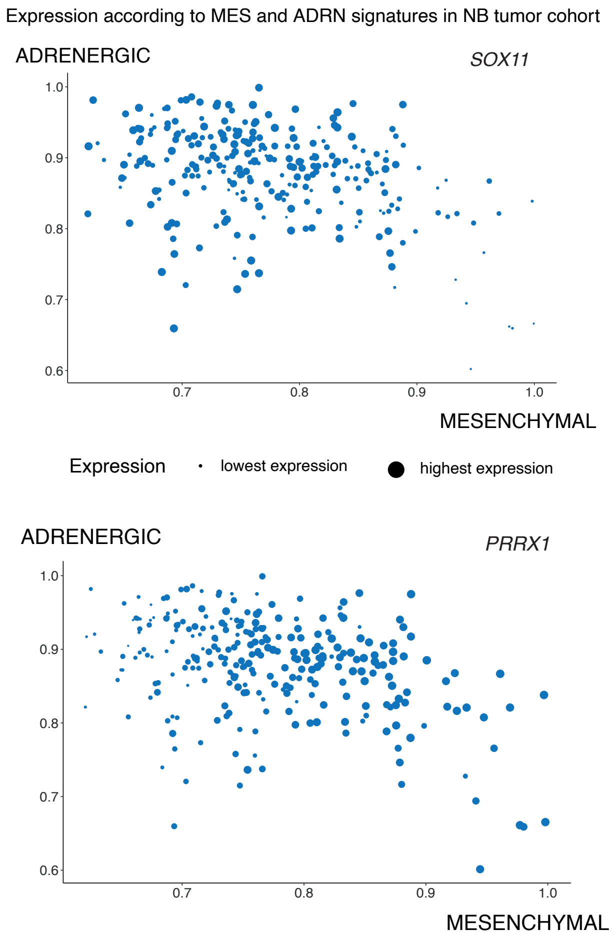

k

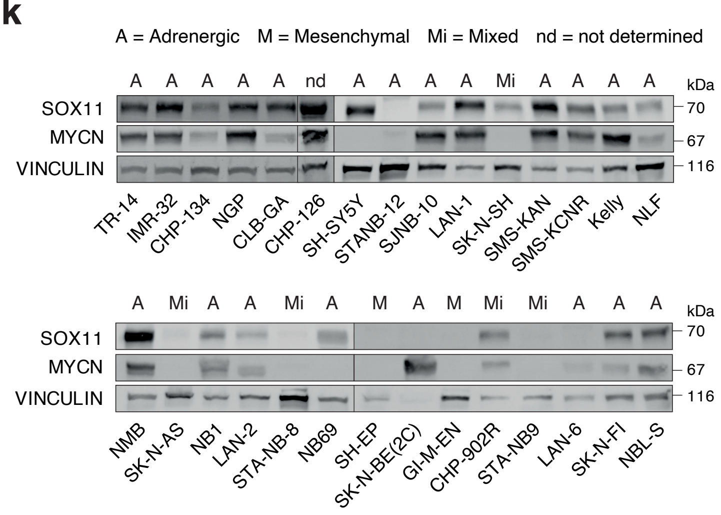

l

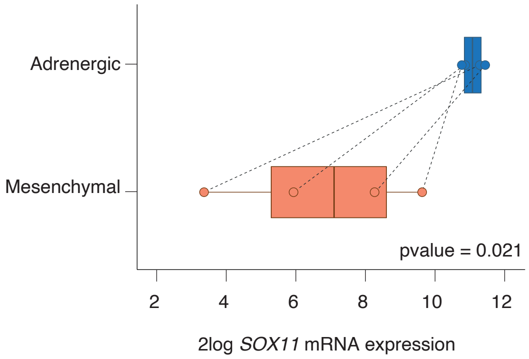

Supplementary Figure 1: Rare focal amplifications and lineage-specific overexpression of *SOX11* in NB

A. Log2 copy number ratio on chr2p (2-18Mb, hg19), encompassing *SOX11* and *MYCN*, for samples depicted in Figure1A.
B. Kaplan-Meier analysis with high or low *SOX11* expression (highest quartile cut-off) of 276 (GSE85047[1], 207 patients low and 69 patients with high expression) and 498 neuroblastoma patients (GSE62564[2], 373 patients low and 125 patients with high expression).
C. IHC for SOX11 with two SOX11 antibodies on TMA of NB tumors[3] (n=71, r=889, p=3.94e-24, two-tailed Spearman correlation). Trend line shows 95% confidence interval.
D. S*OX11* (log2) expression in *MYCN*-amplified (MNA) high-risk versus *MYCN* non-amplified (MNoA) high-risk and low-risk tumors (GSE62564[2], n=498) (ANOVA-test with two-tailed Tukey-test).
E. Copy number ratio (log2) of *SOX11* in CCLE database.
F. Genome-wide methylation profile evaluated by reduced representation bisulfite sequencing (RRBS) of *SOX11* in CCLE dataset.
G. *SOX11* (log2) expression in neuroblasts compared to cortex tissue in foetal adrenal glands, during induced differentiation of human sympatho-adrenal precursor cells along the sympatho-adrenal lineage and in NB tumors (Van Haver et al., in preparation).
   For Supplementary Figure 1e-f, NB tumors are shown in blue, other entities in grey. For Supplementary Figure 1e-g, brackets indicated number of replicates.
H. *SOX11* (log2) expression in tumors of *ALK*, *LIN28B*, *LSL-MYCN* and *TH-MYCN* mouse models and mouse adrenal glands[4].
I. *SOX11* (log2) mRNA expression in *TH-MYCN*$^{+/+}$ mice (blue) and normal sympathetic ganglia (grey) (E-MTAB-3247[5]). Standard deviation of 4 biological replicates is shown (moderated two-tailed t-test of Limma Voom, adj.Pvalue=7.63e-6).
J. Activity score (rankSum) for adrenergic (ADRN) and mesenchymal (MES) signature[6] in 283 NB tumors (GSE85047[1]). *SOX11* and *PRRX1* expression is represented by size.
   Boxplots in Figure D, E and I show 1st quartile to 3rd quartile and median. Whiskers show outer quartiles maximized at 1.5 times the size of the box.
K. MYCN, SOX11 and Vinculin protein levels in NB cell lines, annotated as MES-type (M), MIXED-type (Mi), ADRN-type (A) or not dermined (nd)[6,7], and ADRN/MES signature (Figure 2D). Blots were imaged in parallel and repeated twice with similar results.
L. *SOX11* log2 expression in isogenic adrenergic (blue) and mesenchymal (orange) pairs (n=4, connected by lines, two-tailed t-test pvalue=0.021) (GSE90803[8]).
   For Supplementary Figure 1b-l, Source Data file is provided.

Supplemental Figure 2: *SOX11* is flanked by multiple *cis*-interacting adrenergic specific enhancers

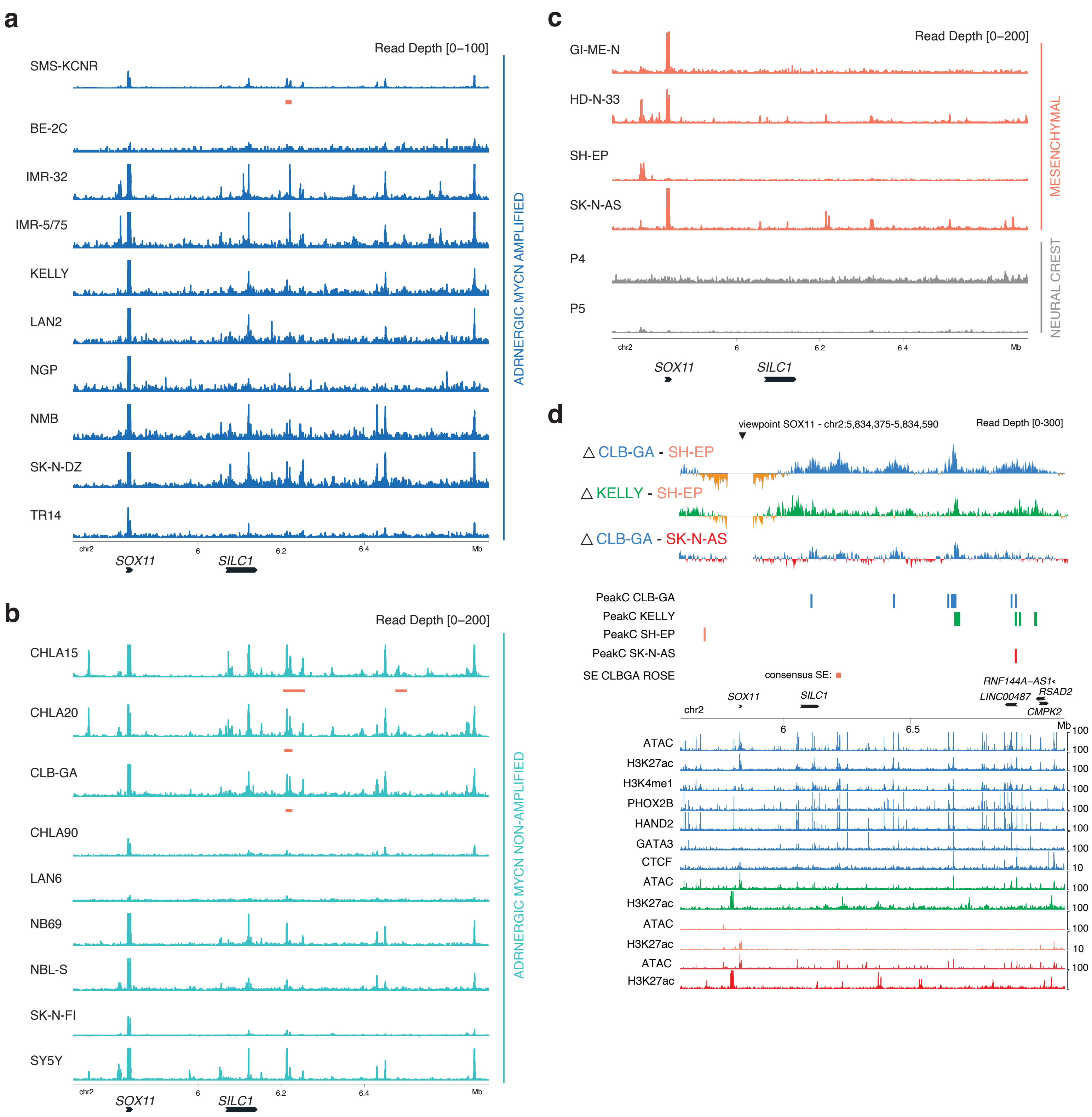

Supplementary Figure 2: *SOX11* is flanked by multiple cis-interacting adrenergic specific enhancers

A. H3K27ac activity for the region immediately downstream of *SOX11* (chr2, 5.7–7.0Mb, hg19) in 10 adrenergic *MYCN* amplified cell lines (SMS-KCNR, BE-2C, IMR-32, IMR-5/75, KELLY, LAN2, NGP, NMB, SK-N-DZ and TR14) (GSE136209)[9].

B. H3K27ac activity for the region immediately downstream of *SOX11* (chr2, 5.7–7.0Mb, hg19) in 9 adrenergic *MYCN* non-amplified cell lines (CHLA15, CHLA20, CLB-GA, CHLA90, LAN6, NB69, NBL-S, SK-N-FI and SY5Y) (GSE136209)[9].

C. H3K27ac activity for the region immediately downstream of *SOX11* (chr2, 5.7–7.0Mb, hg19) in 4 mesenchymal NB cell lines (GI-ME-N, HD-N-33, SH-EP and SK-N-AS) and 2 nonmalignant neural crest cells (P4 and P5) (GSE136209)[9].

   For figure A-C, signal represents RPKM normalised ChIP signal, super-enhancers are annotated using ROSE (orange).

D. 4C-seq analysis of the promoter site and (super-)enhancer region downstream of *SOX11* (chr2, 5.6-7.1Mb, hg19) in the NB cell lines CLB-GA (blue), KELLY (green), SH-EP (orange) and SK-N-AS (red). The viewpoint is located at the *SOX11* transcription start site (cut out 100 kb). Differential track is show that interactions with downstream enhancers and the *SOX11* promoter were present in adrenergic *SOX11* expressing cell lines (CLB-GA and KELLY) and absent in *SOX11*-negative mesenchymal cell lines (SH-EP and SK-N-AS). Interaction peaks called by PeakC are shown underneath 4C-seq data. Published ChIP and ATAC tracks for ATAC (GSE224245), H3K27ac (GSE136209[9]), H3K4me1 (E-MTAB-6570[10]), PHOX2B, HAND2, GATA3 (GSE90683[7]) and CTCF (GSE224245) in CLB-GA, ATAC (GSE138293[11]) and H3K27ac (GSE136209[9]) in KELLY, ATAC (GSE224245) and H3K27ac (GSE189174[12]) in SH-EP and ATAC (GSE138293[11]) and H3K27ac (GSE136209[12]) in SK-NAS. Signal represents log likelihood ratio for the ChIP signal as compared to the input signal (RPM normalised). Super-enhancers (SE) of CLB-GA are annotated using ROSE (orange bar) (GSE136209)[9].

Supplementary Figure 3: SOX11 is a dependency factor in adrenergic NB cells

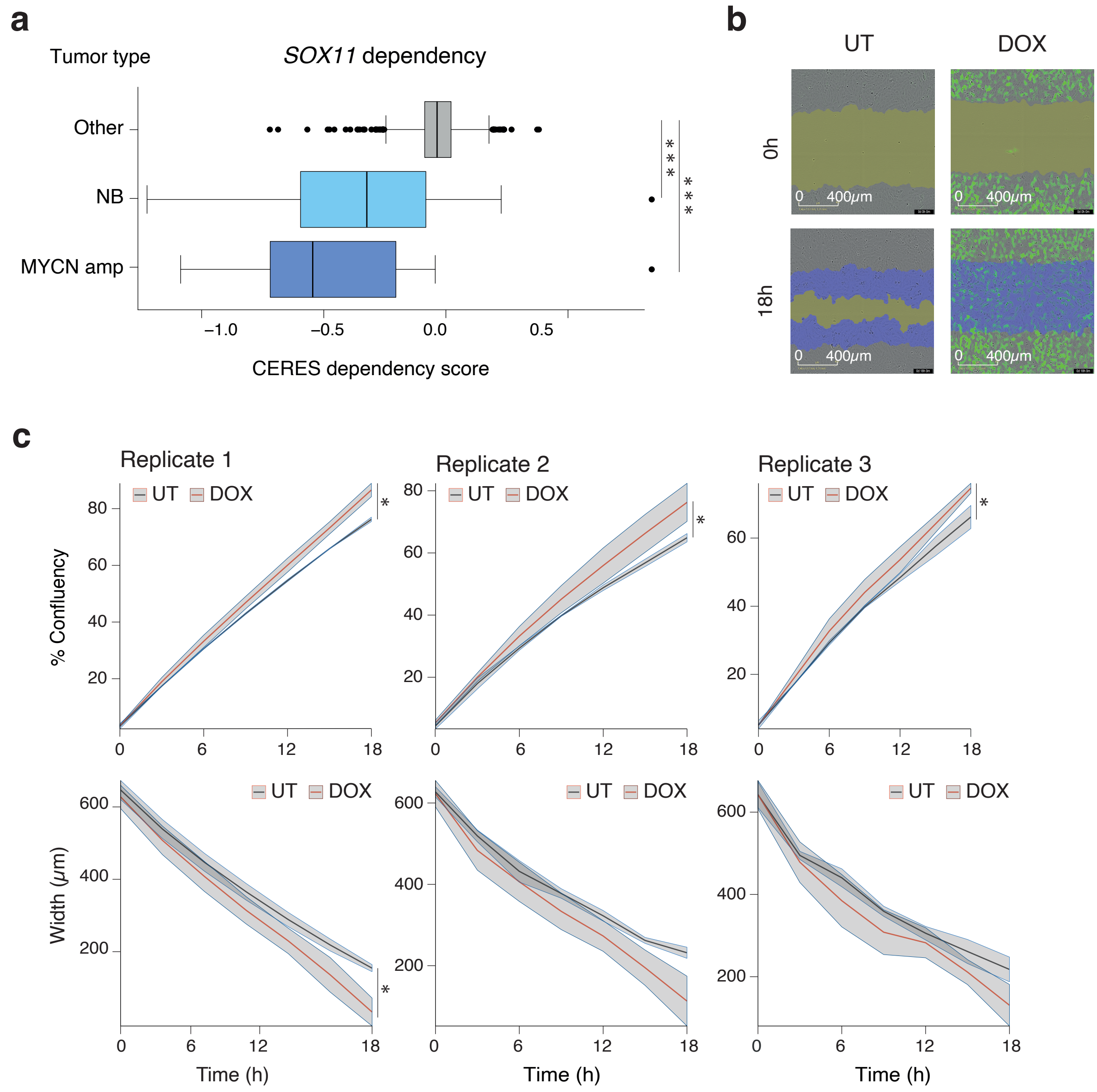

Supplementary Figure 3: SOX11 is a dependency factor in adrenergic NB cells

A. *SOX11* is characterized as a strong selective gene according to a publicly available CRISPR screen in 1086 cell lines (DepMap CRISPR 22Q2 Chronos) with dependency in 25/34 NB cell lines (CERES < -0.1), and significantly selective for NB (p=2.5e-40) and more specifically in 11/15 *MYCN* amplified NB cell lines (p=7.4e-29). Boxplots show the 1st quartile up to the 3rd quartile of the data values and median as a line within the box. Whiskers represent the values of the outer two quartiles maximized at 1.5 times the size of the box. If one or more values outside of the whiskers are present, then this is indicated with a single dot next to the implicated whisker. Figure and citation from https://depmap.org/portal: "Two-group comparisons were performed in parallel across genes/compounds using the Limma R package[13], which uses parametric empirical Bayes methods to pool information across genes when assessing the significance of observed group differences. P-values for each gene are computed from two-tailed empirical Bayes moderated t-statistics. Enriched lineages are those with p-value < 0.0005.

B. Induced wound healing capacity 18h without (UT = untreated) and with (DOX = doxycycline induced expression) *SOX11* overexpression in SH-EP cells. The fluorescence marker ZsGreen is visible when *SOX11* expression is induced. The purple color indicates the migrated cells in the scratch over time, computed with the IncuCyte® ZOOM Software. This experiment was repeated independently three times with similar results. Scalebar represents 400μm.

C. Confluency (%) (up, p=0.016, p=0.036 and p=0.038 respectively) and scratch width (down, p=0.024, p=0.093 and p=0.16 respectively) upon *SOX11* overexpression in SH-EP cells using 3 different monoclonal expansions (3 independent biological replicates). The error bars represent the 95% confidence interval and the average of three independent technical replicates. For statistical testing, a Levine's test was performed in SPSS (v27) at the 1% significance level upon which an independent paired t-test was performed at the 5% significance level.

   For Supplementary Figure 3a and c, Source Data file is provided.

Supplementary Figure 4: The SOX11 regulated transcriptome is involved in epigenetic control, cytoskeleton and neurodevelopment (1 of 2)

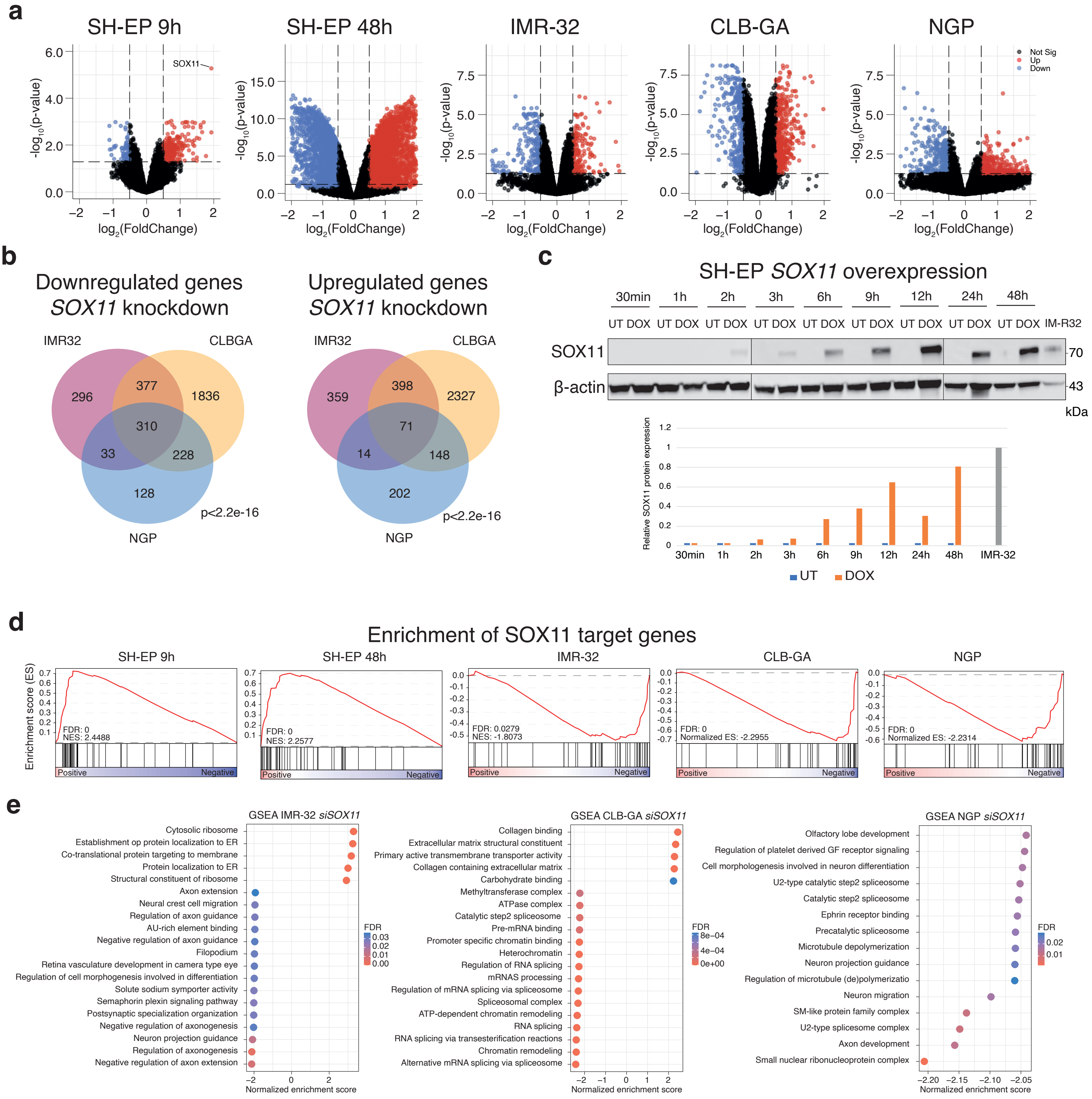

Supplementary Figure 4: The SOX11 regulated transcriptome is involved in epigenetic control, cytoskeleton and neurodevelopment (2 of 2)

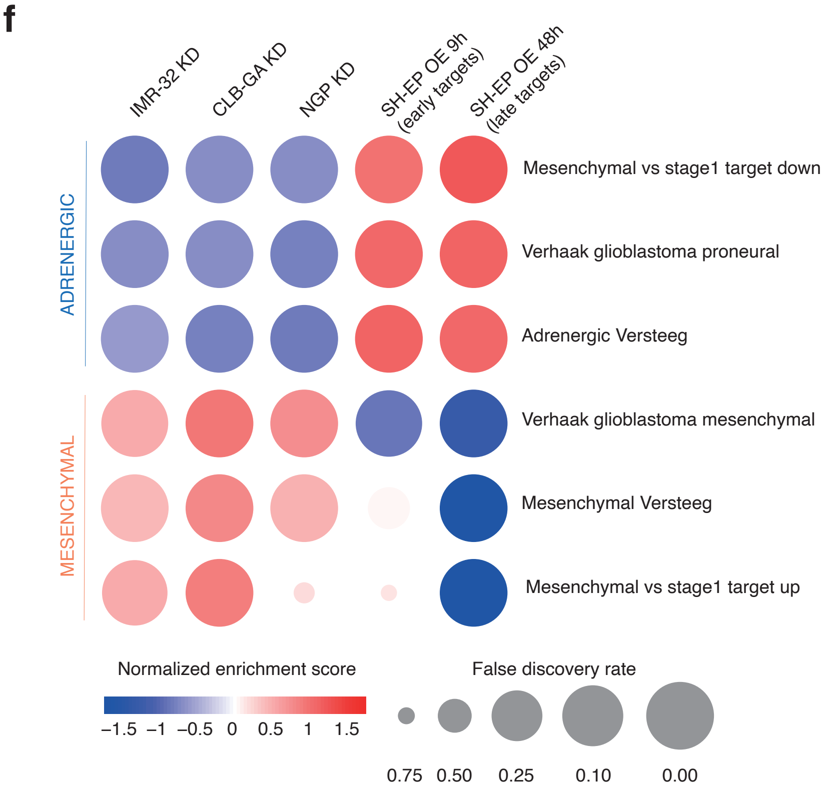

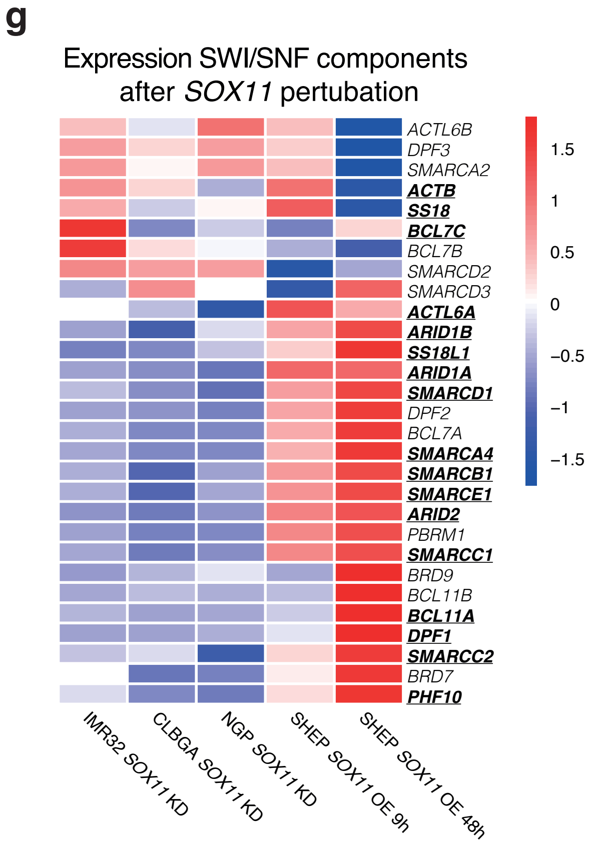

Bold and underlined = bound by SOX11 in IMR-32, CLB-GA, NGP and SH-EP DOX

Supplementary Figure 4: The SOX11 regulated transcriptome is involved in epigenetic control, cytoskeleton and neurodevelopment

A. Volcanoplot showing genes differentially expressed upon *SOX11* overexpression for 9h and 48h in SH-EP and *SOX11* knockdown in IMR-32, CLB-GA and NGP (adj p value < 0.05, log FC > 0.5 or < -0.5). Statistical testing was done using the empirical Bayes quasi-likelihood F-test.
B. Overlap of genes downregulated (left) and upregulated (right) in IMR-32, CLB-GA and NGP upon *SOX11* knockdown for 48h (adj p value < 0.05, two-tailed fisher test p-value<2.2e-16).
C. (top) SOX11 protein levels without (UT = untreated) and with (DOX = doxycycline induced expression) *SOX11* overexpression in SH-EP cells and SOX11 protein levels in IMR-32. Beta-actine is used as loading control. (bottom) Quantification using ImageJ of the relative SOX11 protein expression levels in SH-EP after *SOX11* overexpression compared to SOX11 levels in IMR-32. SOX11 expression levels are normalized to the loading control. Blots are imaged in parallel. The experiment was repeated independently twice with similar results.
D. Enrichment of SOX11 target genes (MsigDB geneset M30174[14]) upon *SOX11* overexpression for 9h and 48h in SH-EP and *SOX11* knockdown in IMR-32, CLB-GA and NGP.
E. Top enriched genesets after doing GSEA analysis (http://www.gsea-msigdb.org/gsea/index.jsp, ontology gene sets C5) for differential genes after knockdown of *SOX11* in IMR-32, CLB-GA and NGP respectively. Depicted is the normalized enrichment score (NES, x-axis) and the false discovery rate (FDR, color).
F. Enrichment of the Proneural and Mesenchymal genesets in glioblastoma[15], the Adrenergic and Mesenchymal genesets in neuroblastoma[6] and the mesenchymal vs NB stage 1[1] genesets in the differential genes upon *SOX11* knockdown in IMR-32, CLB-GA and NGP and *SOX11* overexpression (OE) for 9h and 48h in SH-EP showing normalized enrichment score (color) and false discovery rate (size).
G. Log(FoldChange) of SWI/SNF components upon *SOX11* knockdown in IMR-32, CLB-GA and NGP as well *SOX11* overexpression for 9h and 48h in SH-EP represented in a heatmap. Heatmap color reflects row-wise z-score. Bold and underlined represents genes that are bound by SOX11 in IMR-32, CLB-GA, NGP and SH-EP after *SOX11* overexpression for 48h. For Supplementary Figure 4a, c, and e-g, Source Data file is provided.

Supplementary Figure 5: SOX11 directly regulates multiple major modulators of the epigenome including the SWI/SNF remodeling complex (1 of 2)

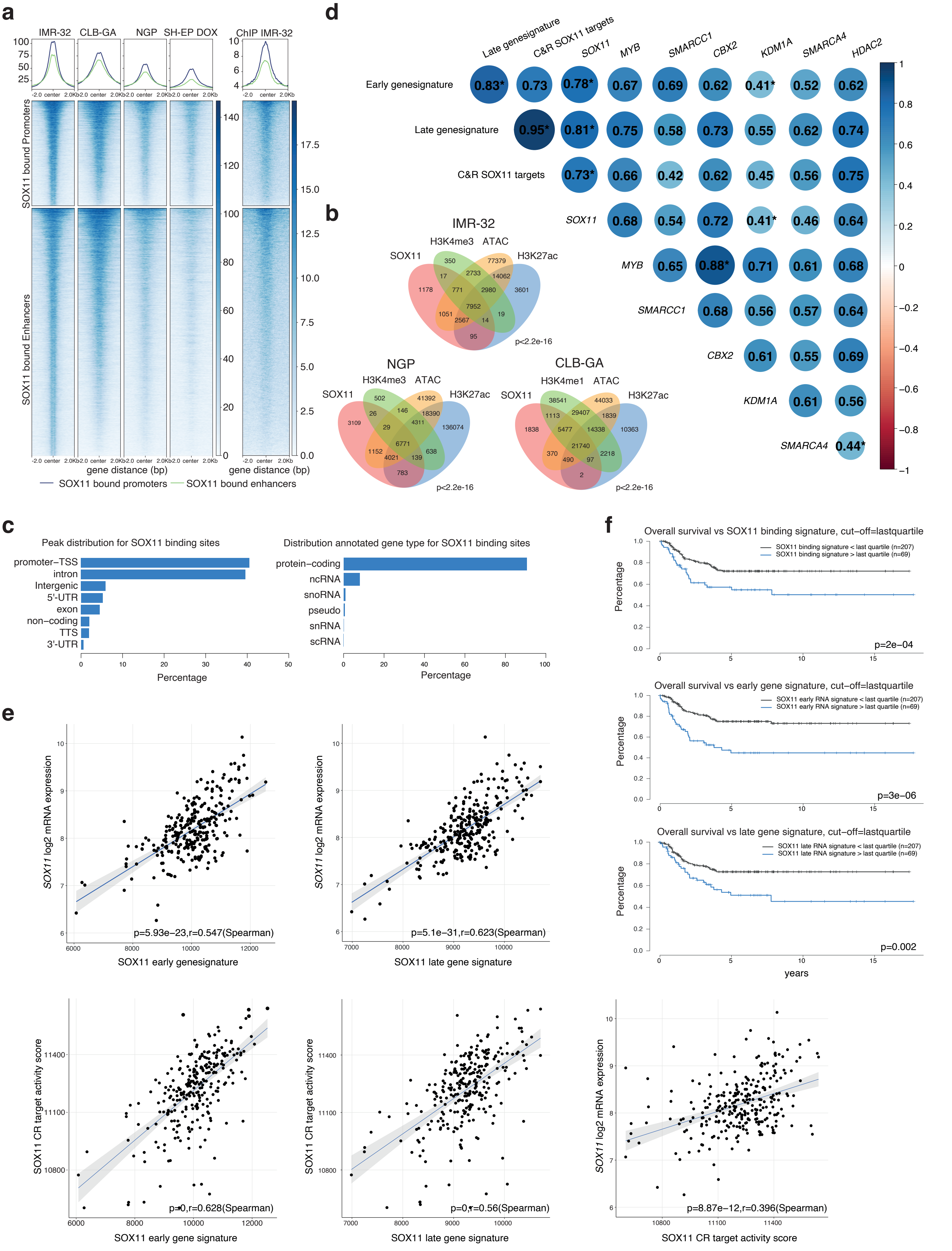

Supplementary Figure 5: SOX11 directly regulates multiple major modulators of the epigenome including the SWI/SNF remodeling complex (2 of 2)

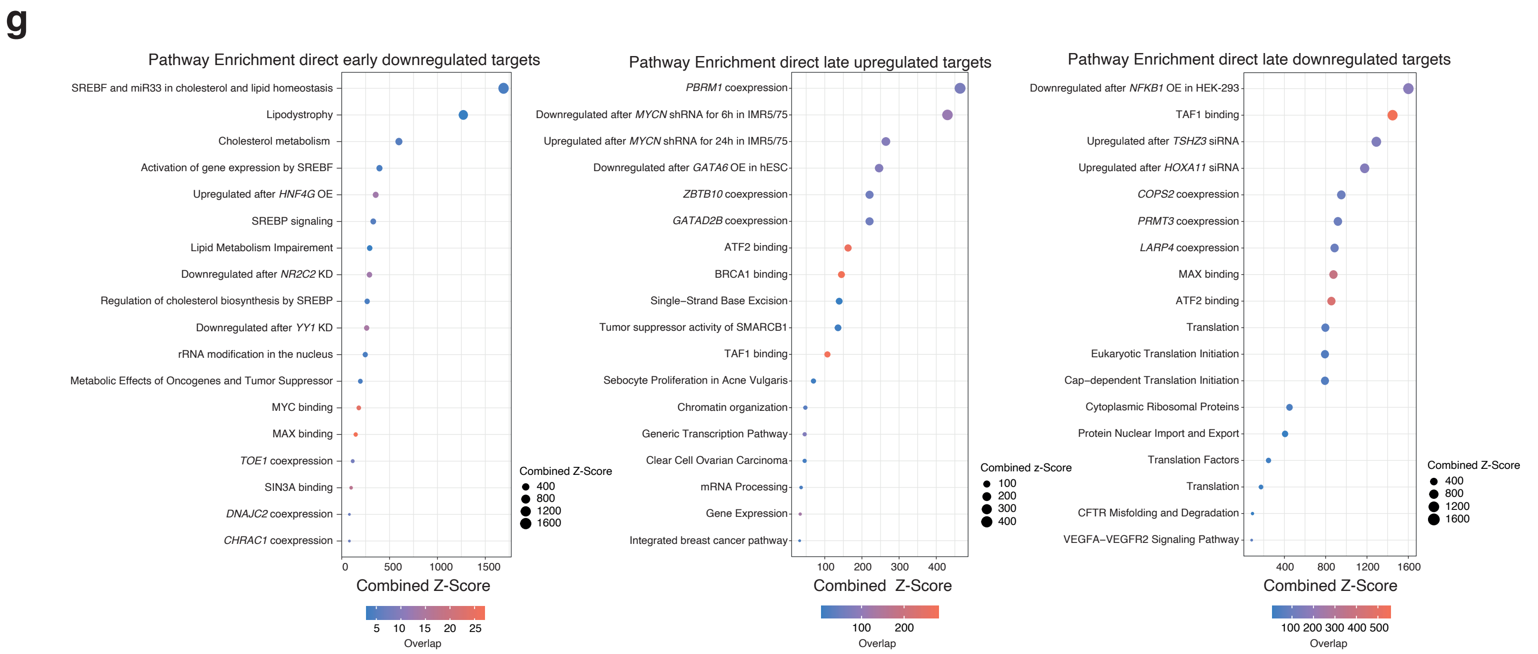

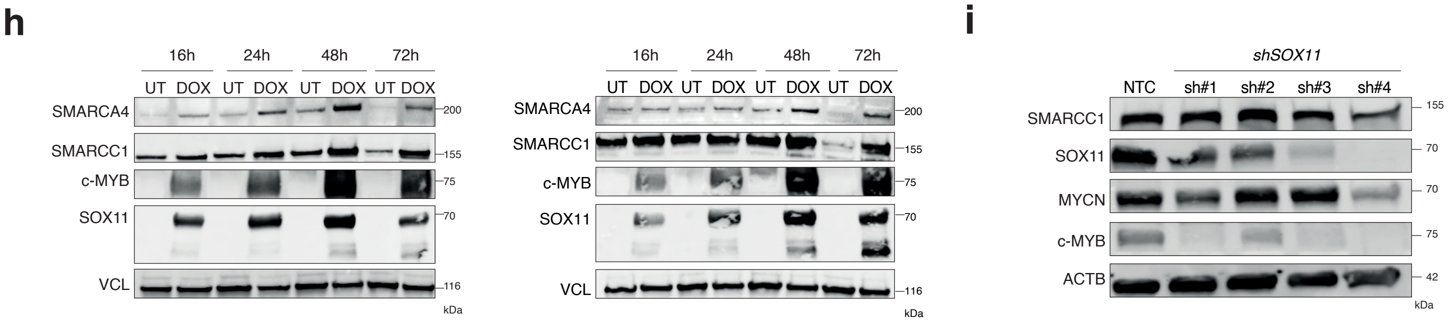

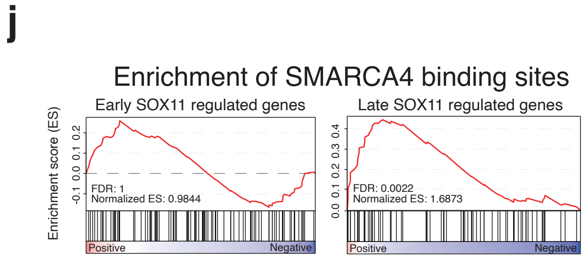

Supplementary Figure 5: SOX11 directly regulates multiple major modulators of the epigenome including the SWI/SNF remodeling complex

A. Heatmap profiles −/+2 kb around summit of SOX11 CUT&RUN peaks in IMR-32, showing SOX11 CUT&RUN data in IMR-32, CLB-GA, NGP and SH-EP cells after *SOX11* overexpression for 48h (SH-EP DOX) and SOX11 ChIP-seq data in IMR-32. Data is grouped for promoters or enhancers (HOMER) and ranked by sums of the peak scores.

B. Overlap (min.overlap = 20 bp) of the SOX11 CUT&RUN and H3K27ac, H3K4me3 (E-MTAB6570[10]), and ATAC peaks (GSE224245) in IMR-32, H3K27ac (GSE136209[9]), H3K4me1 (E-MTAB-6570[10]) and ATAC peaks (GSE224245) in CLB-GA and H3K27ac, H3K4me3 (GSE138314[11]) and ATAC peaks (GSE80151[16]) in NGP (MACS2, qval< 0.05, one-tail fishertest p-value < $2.2e^{-16}$).

C. Peak and gene annotation distribution (HOMER) for common SOX11 CUT&RUN peaks.

D. Correlation of early and late SOX11 gene signature, common SOX11 CUT&RUN targets (rankSum) and ranked expression of *SOX11* and affected genes c-*MYB*, *SMARCC1*, *CBX2, KDM1A, SMARCA4, HDAC2, ARID1A* and *TET1* in 29 NB cell lines (CCLE RNA expression data). Size and color represents two-tailed spearman correlation. Indicated with star is pvalue<0.05.

E. Correlation of activity score (rankSum) of the early and late SOX11 gene signature, common CUT&RUN targets and *SOX11* mRNA expression levels in NB tumors ($n = 283$, GSE85047[1]) showing two-tailed spearman correlation. Trend line shows 95% confidence interval.

F. Correlation of the activity score (rankSum) of low (207) or high (69) SOX11 binding signature (based on CUT&RUN) and early and late SOX11 gene signature with overall survival in NB tumors ($n = 283$, GSE85047[1], Kaplan-Meier).

G. EnrichR analysis[17] for common SOX11 CUT&RUN peaks and early downregulated and late up- and downregulated genes in SH-EP after *SOX11* overexpression for 48h. Depicted is combined Z-score computed by multiplying the log(p-value) (Fisher-exact test) with the zscore of deviation from the expected rank (size), and number of genes that overlap with the enriched genesets (color).

H. SMARCA4, SMARCC1, c-MYB, SOX11 and Vinculin protein levels in SH-EP cells without (UT) and with (DOX) *SOX11* overexpression.

I. SOX11, SMARCC1, c-MYB, MYCN and ACTB expression in NGP cells upon *SOX11* knockdown using 2 different shRNAs and non-targeting control (NTC). Supplementary Figure 5H is repeated twice and Figure 5I is repeated once.

J. Enrichment of top 200 SMARCA4 target genes (defined by ChIP-sequencing[18], GSE134626) in SOX11 early and late regulated genes. For Supplementary Figure5c-g, Source Data file is provided.

Supplementary Figure 6: SOX11 is a core regulatory circuitry transcription factor in adrenergic NB

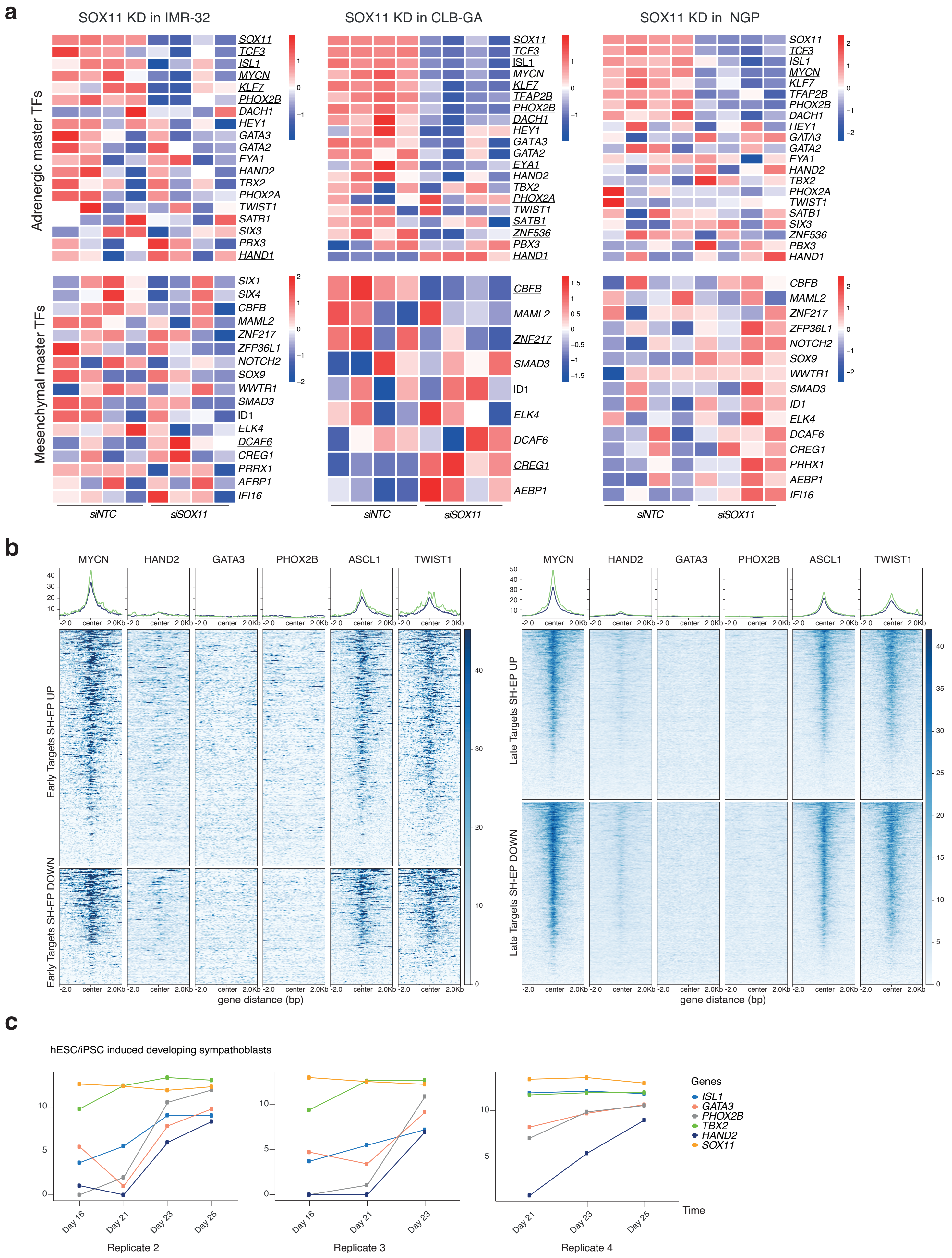

Supplementary Figure 6: SOX11 is a core regulatory circuitry transcription factor in adrenergic NB

A. Log2 mRNA expression of adrenergic CRC members upon *SOX11* knockdown (KD) in IMR-32, CLB-GA and NGP represented in a heatmap. Heatmap color reflects row-wise z-score. Genes that are underlined are differentially expressed.

B. Heatmap profiles −2 kb and +2 kb around the transcription start site of early and late SOX11 targets in SH-EP, subdivided in upregulated and downregulated genes. On these regions HAND2, PHOX2B, GATA3 (GSE90683[7]), MYCN, TWIST1 (GSE94822[19]) and ASCL1 (GSE159613[20]) ChIP data is mapped and ranked according to the sums of the peak scores across all datasets in the heatmap.

C. *ISL1, GATA3, PHOX2B, TBX2, HAND2* and *SOX11* (log2) expression during induced differentiation of hPSC cells along the sympatho-adrenal lineage. Expression levels depicted starting from day 16, when cells are sorted by *SOX10* expression levels indicating cells committed to truncal neural crest cells, and followed-up during SAP development stadia until day 25, for three additional replicates (differentiation tracks).

For Supplementary Figure 6a and c, Source Data file is provided.

Supplementary references

1 Rajbhandari P, Lopez G, Capdevila C, Salvatori B, Yu J, Rodriguez-Barrueco R *et al.* Cross-Cohort Analysis Identifies a TEAD4-MYCN Positive Feedback Loop as the Core Regulatory Element of High-Risk Neuroblastoma. *Cancer Discov* 2018; **8**: 582–599.

2 Su Z, Fang H, Hong H, Shi L, Zhang W, Zhang W *et al.* An investigation of biomarkers derived from legacy microarray data for their utility in the RNA-seq era. *Genome Biol* 2014; **15**: 523.

3 Lammens T, Swerts K, Derycke L, Craemer AD, Brouwer SD, Preter KD *et al.* N-Cadherin in Neuroblastoma Disease: Expression and Clinical Significance. *PLOS ONE* 2012; **7**: e31206.

4 De Wilde B, Beckers A, Lindner S, Kristina A, De Preter K, Depuydt P *et al.* The mutational landscape of MYCN, Lin28b and ALKF1174L driven murine neuroblastoma mimics human disease. *Oncotarget* 2017; **9**: 8334–8349.

5 De Wyn J, Zimmerman MW, Weichert-Leahey N, Nunes C, Cheung BB, Abraham BJ *et al.* MEIS2 Is an Adrenergic Core Regulatory Transcription Factor Involved in Early Initiation of TH-MYCN-Driven Neuroblastoma Formation. *Cancers* 2021; **13**: 4783.

6 van Groningen T, Koster J, Valentijn LJ, Zwijnenburg DA, Akogul N, Hasselt NE *et al.* Neuroblastoma is composed of two super-enhancer-associated differentiation states. *Nat Genet* 2017; **49**: 1261–1266.

7 Boeva V, Louis-Brennetot C, Peltier A, Durand S, Pierre-Eugène C, Raynal V *et al.* Heterogeneity of neuroblastoma cell identity defined by transcriptional circuitries. *Nat Genet* 2017; **49**: 1408–1413.

8 van Groningen T, Koster J, Valentijn LJ, Zwijnenburg DA, Akogul N, Hasselt NE *et al.* Neuroblastoma is composed of two super-enhancer-associated differentiation states. *Nat Genet* 2017; **49**: 1261–1266.

9 Gartlgruber M, Sharma AK, Quintero A, Dreidax D, Jansky S, Park Y-G *et al.* Super enhancers define regulatory subtypes and cell identity in neuroblastoma. *Nat Cancer* 2021; **2**: 114–128.

10 Decaesteker B, Denecker G, Van Neste C, Dolman EM, Van Loocke W, Gartlgruber M *et al.* TBX2 is a neuroblastoma core regulatory circuitry component enhancing MYCN/FOXM1 reactivation of DREAM targets. *Nat Commun* 2018; **9**: 4866.

11 Upton K, Modi A, Patel K, Kendsersky NM, Conkrite KL, Sussman RT *et al.* Epigenomic profiling of neuroblastoma cell lines. *Sci Data* 2020; **7**: 116.

12 Alborzinia H, Flórez AF, Kreth S, Brückner LM, Yildiz U, Gartlgruber M *et al.* MYCN mediates cysteine addiction and sensitizes neuroblastoma to ferroptosis. *Nat Cancer* 2022; **3**: 471–485.

13 Ritchie ME, Phipson B, Wu D, Hu Y, Law CW, Shi W *et al.* limma powers differential expression analyses for RNA-sequencing and microarray studies. *Nucleic Acids Res* 2015; **43**: e47.

14 Kolmykov S, Yevshin I, Kulyashov M, Sharipov R, Kondrakhin Y, Makeev VJ *et al.* GTRD: an integrated view of transcription regulation. *Nucleic Acids Res* 2021; **49**: D104–D111.

15 Verhaak RGW, Hoadley KA, Purdom E, Wang V, Qi Y, Wilkerson MD *et al.* Integrated genomic analysis identifies clinically relevant subtypes of glioblastoma characterized by abnormalities in PDGFRA, IDH1, EGFR, and NF1. *Cancer Cell* 2010; **17**: 98–110.

16 Zeid R, Lawlor MA, Poon E, Reyes JM, Fulciniti M, Lopez MA *et al.* Enhancer invasion shapes MYCN-dependent transcriptional amplification in neuroblastoma. *Nat Genet* 2018; **50**: 515–523.

17 Chen EY, Tan CM, Kou Y, Duan Q, Wang Z, Meirelles GV *et al.* Enrichr: interactive and collaborative HTML5 gene list enrichment analysis tool. *BMC Bioinformatics* 2013; **14**: 128.

18 Shi H, Tao T, Abraham BJ, Durbin AD, Zimmerman MW, Kadoch C *et al.* ARID1A loss in neuroblastoma promotes the adrenergic-to-mesenchymal transition by regulating enhancer-mediated gene expression. *Sci Adv* 2020; **6**: eaaz3440.

19 Durbin AD, Zimmerman MW, Dharia NV, Abraham BJ, Iniguez AB, Weichert-Leahey N *et al.* Selective gene dependencies in MYCN-amplified neuroblastoma include the core transcriptional regulatory circuitry. *Nat Genet* 2018; **50**: 1240–1246.

20 Durbin AD, Wang T, Wimalasena VK, Zimmerman MW, Li D, Dharia NV *et al.* EP300 Selectively Controls the Enhancer Landscape of MYCN-Amplified Neuroblastoma. *Cancer Discov* 2022; **12**: 730–751.
