# Supplementary material for: SOX11 regulates SWI/SNF complex components as member of the adrenergic neuroblastoma core regulatory circuitry
Source: Nat Commun. 2023 Mar 7;14:1267. doi: 10.1038/s41467-023-36735-2 (PMC9992472; doi:10.1038/s41467-023-36735-2)
Supplement: Supplementary file 3 — Description of Additional Supplementary Files [file 41467_2023_36735_MOESM3_ESM.pdf]

Description of Additional Supplementary Files

File Name: Supplementary Data 1
Description: Overview of clinico-genetic parameters of the tumors tested for SOX11 protein expression levels in the tissue micro-array data.

File Name: Supplementary Data 2
Description: Differentially expressed genes (adj.P.Val < 0.05) upon SOX11 knockdown in IMR-32, CLB-GA and NGP cells and SOX11 overexpression in SH-EP cells for 9h and 48h. Statistical testing was done using the empirical Bayes quasi-likelihood F-test.

File Name: Supplementary Data 3
Description: Gene signature of SOX11 early (differential upon SOX11 overexpression at 9h and SOX11 knockdown and correlated expression in 2 NB tumor cohorts) and late (differential upon SOX11 overexpression at 48h but not at 9h and differential upon SOX11 knockdown and correlated expression in 2 NB tumor cohorts) targets.

File Name: Supplementary Data 4
Description: SOX11 CUT&RUN and ChIP-seq targets in IMR-32, CLB-GA, NGP and SH-EP cells after SOX11 overexpression for 48h (MACS2, q.Val < 0.05, gene annotation with homer[38]).

File Name: Supplementary Data 5
Description: : SOX11 CUT&RUN and ChIP-seq motif analysis in IMR-32, CLB-GA, NGP and SH-EP cells after SOX11 overexpression for 48h using homer[38] motif enrichment (known motifs) 200 bp size around peak summit.

File Name: Supplementary Data 6
Description: Cell lines used in the manuscript with the sample ID, origin and MYCN amplification status.
