# Supplementary material for: SOX11 regulates SWI/SNF complex components as member of the adrenergic neuroblastoma core regulatory circuitry
Source: Nat Commun. 2023 Mar 7;14:1267. doi: 10.1038/s41467-023-36735-2 (PMC9992472; doi:10.1038/s41467-023-36735-2)
Supplement: Supplementary file 10 — Reporting Summary [file 41467_2023_36735_MOESM10_ESM.pdf]

nature portfolio

Corresponding author(s): Prof. Dr. Franki Speleman
Dr. Bieke Decaesteker

Last updated by author(s): Jan 9, 2023

Reporting Summary

Nature Portfolio wishes to improve the reproducibility of the work that we publish. This form provides structure for consistency and transparency in reporting. For further information on Nature Portfolio policies, see our Editorial Policies and the Editorial Policy Checklist.

Please do not complete any field with "not applicable" or n/a. Refer to the help text for what text to use if an item is not relevant to your study.
For final submission: please carefully check your responses for accuracy; you will not be able to make changes later.

Statistics

For all statistical analyses, confirm that the following items are present in the figure legend, table legend, main text, or Methods section.

n/a	Confirmed	
☐	☒	The exact sample size (*n*) for each experimental group/condition, given as a discrete number and unit of measurement
☐	☒	A statement on whether measurements were taken from distinct samples or whether the same sample was measured repeatedly
☐	☒	The statistical test(s) used AND whether they are one- or two-sided<br>*Only common tests should be described solely by name; describe more complex techniques in the Methods section.*
☐	☒	A description of all covariates tested
☐	☒	A description of any assumptions or corrections, such as tests of normality and adjustment for multiple comparisons
☐	☒	A full description of the statistical parameters including central tendency (e.g. means) or other basic estimates (e.g. regression coefficient) AND variation (e.g. standard deviation) or associated estimates of uncertainty (e.g. confidence intervals)
☐	☒	For null hypothesis testing, the test statistic (e.g. *F*, *t*, *r*) with confidence intervals, effect sizes, degrees of freedom and *P* value noted<br>*Give P values as exact values whenever suitable.*
☒	☐	For Bayesian analysis, information on the choice of priors and Markov chain Monte Carlo settings
☒	☐	For hierarchical and complex designs, identification of the appropriate level for tests and full reporting of outcomes
☐	☒	Estimates of effect sizes (e.g. Cohen's *d*, Pearson's *r*), indicating how they were calculated

Our web collection on statistics for biologists contains articles on many of the points above.

Software and code

Policy information about availability of computer code

Data collection	Public available fastq files were collected using SRAToolkit (v.2.9.0).
Data analysis	Software used to analyse the data in this study: R (v3.6.1), RStudio (v.1.1.463), the R2 genomics analysis and visualization platform (http://r2.amc.nl/), Vivar (10.1371/journal.pone.0113800), FastQC (v0.11.3), STAR aligner (v2.5.3a), RSEM(v1.2.31), enrichR (10.1186/1471-2105-14-128), Bowtie2 (v.2.3.1), Macs2 (v2.1.0), Homer (v4.10.3), Depmap (CRISPR 22Q2 Chronos), Sushi (v1.32.0), peakC (https://github.com/deWitLab/peakC), IncuCyte® ZOOM Software (v2016B), IncuCyte® S3TM software (v2018B-2019A), SPSS (v27), Modfit LT software package (v6.0), cutadapt (v1.11), edgeR (v3.36.0), limma (v3.503.3), VisionWorks analysis software (v2.0.0), ImageJ (v1.53), TrimGalore (v0.6.5), ChIPpeakAnno (v3.28.1), Deeptool (v3.5.1). Super-enhancers were identified using ROSE (https://bitbucket.org/young_computation/rose).

For manuscripts utilizing custom algorithms or software that are central to the research but not yet described in published literature, software must be made available to editors and reviewers. We strongly encourage code deposition in a community repository (e.g. GitHub). See the Nature Portfolio guidelines for submitting code & software for further information.

Data

Policy information about availability of data

All manuscripts must include a data availability statement. This statement should provide the following information, where applicable:

- Accession codes, unique identifiers, or web links for publicly available datasets
- A description of any restrictions on data availability
- For clinical datasets or third party data, please ensure that the statement adheres to our policy

The RNA-sequencing, CUT&RUN, ChIP-sequencing and ATAC-sequencing datasets generated during this study were deposited in the ArrayExpress database at EMBL-EBI (www.ebi.ac.uk/arrayexpress) with accession numbers: E-MTAB-9340 (RNA-seq IMR-32 siSOX11), E-MTAB-11883 (RNA-seq CLB-GA and NGP siSOX11), E-MTAB-11892 (RNA-seq SHEP 9h SOX11 OE), E-MTAB-9338 (RNA-seq SHEP 48h SOX11 OE), E-MTAB-9464 (ChIP-seq SOX11 IMR-32), E-MTAB-11905 (CUT&RUN SOX11 IMR-32, CLB-GA, NGP and SH-EP 48h SOX11 OE), GSE224245 (CUT&RUN CTCF CLB-GA), GSE224245 (4C-Sequencing SOX11 viewpoint CLB-GA, KELLY, SK-N-AS and SH-EP) and GSE224245 (ATAC-sequencing IMR-32, CLB-GA and SH-EP). Neuroblastoma tumor data that supports the findings of this study are available from the Neuroblastoma Research Consortium (NRC, GSE85047), Su et al. (GSE45547 and GSE62564)29, Depuydt et al. (GSE103123)10, Versteeg et al. (GSE16476)37 and Janoueix-Lerosey et al. (GSE12460)38. Additional tumor data sets were used for glioma (GSE4290), breast cancer (GSE12276), thyroid cancer (GSE2109), lung cancer (GSE2109), prostate cancer (GSE2109), ALL (GSE10609), colon cancer (GSE14333) and ovarian cancer (GSE12172). Cancer cell line information (expression, copy number, methylation and dependency data) is available via the CCLE database (https://depmap.org/portal/). RNA expression data for isogenic NB cell lines used in Supplementary Figure 1L is provided by van Groningen et al. (GSE90803)39. ChIP-seq data are available from: MYCN, TWIST1 (GSE94822), ASCL1 (GSE159613) HAND2, GATA3 and PHOX2B (GSE90683), SMARCA4 (GSE134626). H3K27ac ChIP-seq and RNA-seq expression data from NB cell lines and tumor used in Figure 2 and Supplementary Figure 2 is provided by Gartlgruber et al. (GSE136209)14. Expression data in the TH-MYCN+/+ NB tumor mice model is provided by De Preter et al. (E-MTAB-3247). Additional published data used are H3K27ac (GSE136209), H3K4me1 (E-MTAB-6570) in CLB-GA, ATAC, H3K4me3 and H3K27ac (E-MTAB-6570) in IMR-32, ATAC (GSE80151), H3K4me3 and H3K27ac (GSE138314) in NGP, ATAC (GSE138293) and H3K27ac (GSE136209) in KELLY, H3K27ac (GSE189174) in SH-EP and ATAC (GSE138293) and H3K27ac (GSE136209) in SK-N-AS.

Human research participants

Policy information about studies involving human research participants and Sex and Gender in Research.

Reporting on sex and gender	Sex of participants was determined based on biological assignment of sex/gender at birth. No difference was observed in the expression and molecular function of SOX11 in female and male neuroblastoma patients and cell lines. Hence sex and gender were not considered in this study.
Population characteristics	Human patients with a histopathologically confirmed neuroblastoma (n=68, age range 0-13 years) were included in the study.
Recruitment	For the generation of the TMA, available tumor samples were collected from the biobank thus therefore no active patient recruitment was done for this study.
Ethics oversight	Patient sample collection of NB tumor used for tissue micro-array (TMA) was approved by the ethical committee (Ethical Committee Ghent University Hospital, EC/208-2006/Svdm) and written informed consent was obtained from the patients and/or their parents. All other datasets involving patient data is publicly available and information regarding ethical consent can be found in the linked accession codes and publications.

Note that full information on the approval of the study protocol must also be provided in the manuscript.

Field-specific reporting

Please select the one below that is the best fit for your research. If you are not sure, read the appropriate sections before making your selection.

☒ Life sciences ☐ Behavioural & social sciences ☐ Ecological, evolutionary & environmental sciences

For a reference copy of the document with all sections, see nature.com/documents/nr-reporting-summary-flat.pdf

Life sciences study design

All studies must disclose on these points even when the disclosure is negative.

Sample size	When necessary sample size is (n=) is included on the figure and/or in the legend.
Data exclusions	For Kaplan-Meier analysis, datapoints were omitted from the dataset when no survival d ata were available.<br>No samples from other assays were excluded from the analysis.
Replication	For cell cycle analysis, incucyte proliferation, scratch wound assay and western blot, experiments were completed with 3 biological replicates, the mininum required to perform statistical analysis. For cell cycle analysis, the representative 3 biological replicates are shown by individual dots. For colony formation capacity, 1 biological replicate was shown for each cell line and three biological replicates were performed for statistical analysis. For scratch wound assay, images of one biological 1 biological replicate was shown and three biological replicates were

	performed for statistical analysis. Technical replicates representing the same cells at the same time were included to estimate the mean for every biological replicate, but not used for statistical analysis and not included in the total number of replicates (n=).
Randomization	Experimental groups were defined based on appropriate biological and technical controls. No animal or human participants were used in this study.
Blinding	Incucyte poliferation, scratch wound assay and colony formation assays were analyzed by two different individuals in a blinded fashion.

Behavioural & social sciences study design

All studies must disclose on these points even when the disclosure is negative.

Study description	*Briefly describe the study type including whether data are quantitative, qualitative, or mixed-methods (e.g. qualitative cross-sectional, quantitative experimental, mixed-methods case study).*
Research sample	*State the research sample (e.g. Harvard university undergraduates, villagers in rural India) and provide relevant demographic information (e.g. age, sex) and indicate whether the sample is representative. Provide a rationale for the study sample chosen. For studies involving existing datasets, please describe the dataset and source.*
Sampling strategy	*Describe the sampling procedure (e.g. random, snowball, stratified, convenience). Describe the statistical methods that were used to predetermine sample size OR if no sample-size calculation was performed, describe how sample sizes were chosen and provide a rationale for why these sample sizes are sufficient. For qualitative data, please indicate whether data saturation was considered, and what criteria were used to decide that no further sampling was needed.*
Data collection	*Provide details about the data collection procedure, including the instruments or devices used to record the data (e.g. pen and paper, computer, eye tracker, video or audio equipment) whether anyone was present besides the participant(s) and the researcher, and whether the researcher was blind to experimental condition and/or the study hypothesis during data collection.*
Timing	*Indicate the start and stop dates of data collection. If there is a gap between collection periods, state the dates for each sample cohort.*
Data exclusions	*If no data were excluded from the analyses, state so OR if data were excluded, provide the exact number of exclusions and the rationale behind them, indicating whether exclusion criteria were pre-established.*
Non-participation	*State how many participants dropped out/declined participation and the reason(s) given OR provide response rate OR state that no participants dropped out/declined participation.*
Randomization	*If participants were not allocated into experimental groups, state so OR describe how participants were allocated to groups, and if allocation was not random, describe how covariates were controlled.*

Ecological, evolutionary & environmental sciences study design

All studies must disclose on these points even when the disclosure is negative.

Study description	*Briefly describe the study. For quantitative data include treatment factors and interactions, design structure (e.g. factorial, nested, hierarchical), nature and number of experimental units and replicates.*
Research sample	*Describe the research sample (e.g. a group of tagged Passer domesticus, all Stenocereus thurberi within Organ Pipe Cactus National Monument), and provide a rationale for the sample choice. When relevant, describe the organism taxa, source, sex, age range and any manipulations. State what population the sample is meant to represent when applicable. For studies involving existing datasets, describe the data and its source.*
Sampling strategy	*Note the sampling procedure. Describe the statistical methods that were used to predetermine sample size OR if no sample-size calculation was performed, describe how sample sizes were chosen and provide a rationale for why these sample sizes are sufficient.*
Data collection	*Describe the data collection procedure, including who recorded the data and how.*
Timing and spatial scale	*Indicate the start and stop dates of data collection, noting the frequency and periodicity of sampling and providing a rationale for these choices. If there is a gap between collection periods, state the dates for each sample cohort. Specify the spatial scale from which the data are taken*
Data exclusions	*If no data were excluded from the analyses, state so OR if data were excluded, describe the exclusions and the rationale behind them, indicating whether exclusion criteria were pre-established.*
Reproducibility	*Describe the measures taken to verify the reproducibility of experimental findings. For each experiment, note whether any attempts to repeat the experiment failed OR state that all attempts to repeat the experiment were successful.*
Randomization	*Describe how samples/organisms/participants were allocated into groups. If allocation was not random, describe how covariates were controlled. If this is not relevant to your study, explain why.*

Blinding

Describe the extent of blinding used during data acquisition and analysis. If blinding was not possible, describe why OR explain why blinding was not relevant to your study.

Did the study involve field work? ☐ Yes ☐ No

Field work, collection and transport

Field conditions

Describe the study conditions for field work, providing relevant parameters (e.g. temperature, rainfall).

Location

State the location of the sampling or experiment, providing relevant parameters (e.g. latitude and longitude, elevation, water depth).

Access & import/export

Describe the efforts you have made to access habitats and to collect and import/export your samples in a responsible manner and in compliance with local, national and international laws, noting any permits that were obtained (give the name of the issuing authority, the date of issue, and any identifying information).

Disturbance

Describe any disturbance caused by the study and how it was minimized.

Reporting for specific materials, systems and methods

We require information from authors about some types of materials, experimental systems and methods used in many studies. Here, indicate whether each material, system or method listed is relevant to your study. If you are not sure if a list item applies to your research, read the appropriate section before selecting a response.

Materials & experimental systems

n/a	Involved in the study	
☐	☒	Antibodies
☐	☒	Eukaryotic cell lines
☒	☐	Palaeontology and archaeology
☒	☐	Animals and other organisms
☒	☐	Clinical data
☒	☐	Dual use research of concern

Methods

n/a	Involved in the study	
☐	☒	ChIP-seq
☒	☐	Flow cytometry
☒	☐	MRI-based neuroimaging

Antibodies

Antibodies used

Primary antibodies for TMA and western blot: SOX11-C1 antibody (From Prof. Sara Ek, Lund University, Nordström et al., BMC cancer, 2012, 1:300 dilution), SOX11
antibody (ILM3823-C01, Klinipath,1:50 dilution), SOX11-PAb (see below, 1:1000 dilution), anti-c-MYB antibody (12319S, Cell Signaling, 1:1000 dilution), anti-MYCN antibody (SC-53993, Santa Cruz 1:1,000 dilution), anti-SMARCC1 antibody (11956S, Cell Signaling 1:1,000), and anti-SMARCA4 antibody (3508S, Cell Signaling, 1:500). Secondary antibodies for TMA and western blot: HRPlabeled
anti-rabbit (7074S, Cell Signalling, 1:10,000) and anti-mouse (7076P2, Cell Signalling, 1:10,000) antibodies. Antibodies against Vinculin (V9131; Sigma-Aldrich, 1:10,000), alpha-Tubulin (T5168, Sigma-Aldrich, 1:10,000) or bèta actine (A2228; Sigma-Aldrich, 1:10,000) were used as loading control. The rabbit polyclonal antibody, SOX11-PAb, was custom made (Absea biotechnology, China) against the immunogenic peptide p-SOX11C-term
DDDDDDDDDELQLQIKQEPDEEDEEPPHQQLLQPPGQQPSQLLRRYNVAKVPASPTLSSSAESPEGASLYDEVRAGATSGAGGGSRLYYSFKNITKQHPP
PLAQPALSPASSRSVSTSSS and used for western blot and chromatin immunoprecipitation for SOX11. For CUT&RUN, anti-SOX11 antibody (HPA000536, Atlas Antibodies), anti-IgG goat antibody (sc-2028, Santa Cruz Biotechnology) and anti-CTCF antibody (07-729, merck) was used.

Validation

Antibodies were selected based on validation described on the manufacturer's website and existing citations and/or were validated in the lab by western blot with total protein lysates collected from different neuroblastoma cell lines. All catalogue numbers are listed in the section "Methods", subsection "Tissue Micro-array" or "Western blot analysis".
• SOX11-C1 antibody (From Prof. Sara Ek, Lund University, Nordström et al., BMC cancer, 2012) for validation see Figure 3A-B and Supplementary Fig. 1K and 5H-I
• SOX11-antibody (ILM3823-C01, Klinipath) ▯ has been validated for immunohistochemistry in mantle cell lymphoma: 10.3324/haematol.2009.010264
• SOX11-PAb (see section ab used) ▯ validation see Supplementary Fig. 4C
• anti-c-MYB antibody (12319S, Cell Signaling) validated for WB by Cell signaling
• anti-MYCN antibody (SC-53993, Santa Cruz) ▯ validated by santa cruz by Western blot analysis of N-Myc expression in NB cell lines IMR-32 and SH-SY5Y whole cell lysates and by additional citations eg 10.1038/s43018-022-00355-4
• anti-SMARCC1 antibody (11956S, Cell Signaling) ▯ validated for WB by Cell signaling
• anti-SMARCA4 antibody (3508S, Cell Signaling) ▯ validated for WB by Cell signaling

• HRPlabeled anti-rabbit (7074S, Cell Signalling) ⍰ validated for WB by Cell signaling
• HRPlabeled anti-mouse (7076P2, Cell Signalling) ⍰ validated for WB by Cell signaling
• Vinculin (V9131; Sigma-Aldrich) ⍰ validated for WB by sigma Aldrich
• alpha-Tubulin (T5168, Sigma-Aldrich) ⍰ validated for WB by sigma Aldrich
• bèta actine (A2228; Sigma-Aldrich) ⍰ validated for WB by sigma Aldrich
• anti-SOX11 antibody (HPA000536, Atlas Antibodies) -> Validated by Atlas Antibodies with IHC tissue array of 44 normal human tissues and 20 of the most common cancer type tissues and protein array of 346 human recombinant protein fragments.
• anti-IgG goat antibody (sc-2028, Santa Cruz Biotechnology) ⍰ has been validated for ChIP: 10.1038/s41588-018-0085-0

Eukaryotic cell lines

Policy information about cell lines and Sex and Gender in Research

Cell line source(s): The source of each cell line used is stated in Supplementary Table 6. The COG-N-373 cell line (cell line with SOX11 focal amplification) was a kindly gift from P. Reynolds and M. Hogarty.

Authentication: All cell lines were authenticated by STR genotyping. Reported on Manuscript page 18, section "Materials & Methods", subsection "Samples and cell lines."

Mycoplasma contamination: All cell lines were tested for mycoplasma on a monthly basis and confirmed negative. Reported on Manuscript page 18, section "Materials & Methods", subsection "Samples and cell lines."

Commonly misidentified lines (See ICLAC register): No commonly misidentified cell lines are used in this study

Palaeontology and Archaeology

Specimen provenance: *Provide provenance information for specimens and describe permits that were obtained for the work (including the name of the issuing authority, the date of issue, and any identifying information). Permits should encompass collection and, where applicable, export.*

Specimen deposition: *Indicate where the specimens have been deposited to permit free access by other researchers.*

Dating methods: *If new dates are provided, describe how they were obtained (e.g. collection, storage, sample pretreatment and measurement), where they were obtained (i.e. lab name), the calibration program and the protocol for quality assurance OR state that no new dates are provided.*

☐ Tick this box to confirm that the raw and calibrated dates are available in the paper or in Supplementary Information.

Ethics oversight: *Identify the organization(s) that approved or provided guidance on the study protocol, OR state that no ethical approval or guidance was required and explain why not.*

Note that full information on the approval of the study protocol must also be provided in the manuscript.

Animals and other research organisms

Policy information about studies involving animals; ARRIVE guidelines recommended for reporting animal research, and Sex and Gender in Research

Laboratory animals: *For laboratory animals, report species, strain and age OR state that the study did not involve laboratory animals.*

Wild animals: *Provide details on animals observed in or captured in the field; report species and age where possible. Describe how animals were caught and transported and what happened to captive animals after the study (if killed, explain why and describe method; if released, say where and when) OR state that the study did not involve wild animals.*

Reporting on sex: *Indicate if findings apply to only one sex; describe whether sex was considered in study design, methods used for assigning sex. Provide data disaggregated for sex where this information has been collected in the source data as appropriate; provide overall numbers in this Reporting Summary. Please state if this information has not been collected. Report sex-based analyses where performed, justify reasons for lack of sex-based analysis.*

Field-collected samples: *For laboratory work with field-collected samples, describe all relevant parameters such as housing, maintenance, temperature, photoperiod and end-of-experiment protocol OR state that the study did not involve samples collected from the field.*

Ethics oversight: *Identify the organization(s) that approved or provided guidance on the study protocol, OR state that no ethical approval or guidance was required and explain why not.*

Note that full information on the approval of the study protocol must also be provided in the manuscript.

Clinical data

Policy information about clinical studies

All manuscripts should comply with the ICMJE guidelines for publication of clinical research and a completed CONSORT checklist must be included with all submissions.

Clinical trial registration	*Provide the trial registration number from ClinicalTrials.gov or an equivalent agency.*
Study protocol	*Note where the full trial protocol can be accessed OR if not available, explain why.*
Data collection	*Describe the settings and locales of data collection, noting the time periods of recruitment and data collection.*
Outcomes	*Describe how you pre-defined primary and secondary outcome measures and how you assessed these measures.*

Dual use research of concern

Policy information about dual use research of concern

Hazards

Could the accidental, deliberate or reckless misuse of agents or technologies generated in the work, or the application of information presented in the manuscript, pose a threat to:

No	Yes	
☐	☐	Public health
☐	☐	National security
☐	☐	Crops and/or livestock
☐	☐	Ecosystems
☐	☐	Any other significant area

Experiments of concern

Does the work involve any of these experiments of concern:

No	Yes	
☐	☐	Demonstrate how to render a vaccine ineffective
☐	☐	Confer resistance to therapeutically useful antibiotics or antiviral agents
☐	☐	Enhance the virulence of a pathogen or render a nonpathogen virulent
☐	☐	Increase transmissibility of a pathogen
☐	☐	Alter the host range of a pathogen
☐	☐	Enable evasion of diagnostic/detection modalities
☐	☐	Enable the weaponization of a biological agent or toxin
☐	☐	Any other potentially harmful combination of experiments and agents

ChIP-seq

Data deposition

☒ Confirm that both raw and final processed data have been deposited in a public database such as GEO.

☒ Confirm that you have deposited or provided access to graph files (e.g. BED files) for the called peaks.

Data access links<br>*May remain private before publication.*	ChIP: E-MTAB-9464: https://www.ebi.ac.uk/arrayexpress/experiments/E-MTAB-9464<br>CUT&RUN: E-MTAB-11905: https://www.ebi.ac.uk/arrayexpress/experiments/E-MTAB-11905
Files in database submission	ChIP: fIMR32-INPUT-_S3_L001_R1_001.fastq.gz, fIMR32-INPUT-_S3_L002_R1_001.fastq.gz, fIMR32-INPUT-_S3_L003_R1_001.fastq.gz, fIMR32-INPUT-_S3_L004_R1_001.fastq.gz, gIMR32-SOX11-_S2_L001_R1_001.fastq.gz, gIMR32-SOX11-_S2_L001_R1_002.fastq.gz, gIMR32-SOX11-_S2_L003_R1_001.fastq.gz, gIMR32-SOX11-_S2_L004_R1_001.fastq.gz<br>CUT&RUN: CLBGA_IgG_R1.fastq.gz, CLBGA_IgG_R2.fastq.gz, CLBGA_SOX11_R1.fastq.gz, CLBGA_SOX11_R2.fastq.gz, IMR32_IgG_R1.fastq.gz, IMR32_IgG_R2.fastq.gz, IMR32_SOX11_R1.fastq.gz, IMR32_SOX11_R2.fastq.gz, NGP_IgG_R1.fastq.gz, NGP_IgG_R2.fastq.gz, NGP_SOX11_R1.fastq.gz, NGP_SOX11_R2.fastq.gz, SHEPplusDOX_IgG_R1.fastq.gz, SHEPplusDOX_IgG_R2.fastq.gz, SHEPplusDOX_SOX11_R1.fastq.gz, SHEPplusDOX_SOX11_R2.fastq.gz
Genome browser session<br>(e.g. UCSC)	

For reviewer access: https://www.ebi.ac.uk/arrayexpress/ -
RNA-seq: IMR-32 siSOX11 48h = Username: Reviewer_E-MTAB-9340 with Password: aRcfxsvm
RNA-seq: CLB-GA siSOX11 48h = Username: Reviewer_E-MTAB-11883 with Password: cedww7fE
RNA-seq: NGP SH-EP SOX11 OE 9h = Username: Reviewer_E-MTAB-11892 with Password: 5rlkhypa
RNA-seq: SH-EP SOX11 OE 48h = Username: Reviewer_E-MTAB-9338 with Password: aRcfxsvm
ChIP-seq: IMR-32 SOX11 IP = Username: Reviewer_E-MTAB-9464 with Password: pXXVNC8T
CUT&RUN: IMR-32, CLB-GA, NGP, SH-EP SOX11 OE 48h SOX11 IP = Username: Reviewer_E-MTAB-11905 with Password: ecn9sxz6

Methodology

Replicates	For SOX11 CUT&RUN and SOX11 ChIP we have one biological replicate per cell line.
Sequencing depth	IMR-32 SOX11 ChIP: Total mapped reads:75269021, Uniqually mapped reads: 58444500, Read length: 75 bp, single-end<br>IMR-32 INPUT ChIP: Total mapped reads: 46283806, Uniqually mapped reads: 38101144, Read length: 75 bp, single-end<br>IMR-32 SOX11 CUT&RUN: Total mapped reads: 9055406, Uniqually mapped reads: 6399393, Read length: 75 bp, pair-end<br>IMR-32 IgG CUT&RUN: Total mapped reads: 8941165, Uniqually mapped reads: 5925768 Read length: 75 bp, pair-end<br>CLB-GA SOX11 CUT&RUN: Total mapped reads: 9071586, Uniqually mapped reads: 6736893 Read length: 75 bp, pair-end<br>CLB-GA IgG CUT&RUN: Total mapped reads: 8633535, Uniqually mapped reads: 594113, Read length: 75 bp, pair-end<br>NGP SOX11 CUT&RUN: Total mapped reads: 9121804, Uniqually mapped reads: 6603935, Read length: 75 bp, pair-end<br>NGP IgG CUT&RUN: Total mapped reads: 9118638, Uniqually mapped reads: 5897926, Read length: 75 bp, pair-end<br>SH-EP SOX11 OE SOX11 CUT&RUN: Total mapped reads: 8509115, Uniqually mapped reads: 5373607, Read length: 75 bp, pair-end<br>SH-EP SOX11 OE IgG CUT&RUN: Total mapped reads: 8247565, Uniqually mapped reads: 5869023, Read length: 75 bp, pair-end
Antibodies	CUT&RUN: anti-SOX11 antibody (HPA000536, Atlas Antibodies) and anti-IgG goat antibody (sc-2028, Santa Cruz Biotechnology) was used.<br>ChIP: The rabbit polyclonal antibody, SOX11-PAb, was custom made (Absea biotechnology, China) against the immunogenic peptide p-SOX11C-term<br>DDDDDDDDDELQLQIKQEPDEEDEEPPHQQLLQPPGQQPSQLLRRYNVAKVPASPTLSSSAESPEGASLYDEVRAGATSGAGGGSRLYYSFKNITKQHPPPLAQPALSPASSRSVSTSSS
Peak calling parameters	Read mapping was done with bowtie2 and index hg19.<br>Binding sites were identified using macs2, comparing peak enrichment over input controls, with -q cut-off value of 0.05
Data quality	SOX11 ChIP-seq in IMR-32 cells: number of peaks < FDR 0.05: 3105<br>SOX11 CUT&RUN in IMR-32 cells: number of peaks < FDR 0.05: 14907<br>SOX11 CUT&RUN in CLB-GA cells: number of peaks < FDR 0.05: 38926<br>SOX11 CUT&RUN in NGP cells: number of peaks < FDR 0.05: 17592<br>SOX11 CUT&RUN in SH-EP SOX11 OE cells: number of peaks < FDR 0.05: 17774
Software	Prior to mapping to the human reference genome (GRCh37/hg19) with bowtie2, quality of the raw sequencing data was evaluated using FastQC and adapter dimers were removed using cutadapt. Quality of aligned reads were filtered using min MAPQ 30 and reads with known low sequencing confidence were removed using Encode Blacklist regions. For CUT&RUN, because of oversequencing reads were subsampled, and mapping was done with 10M reads (recommended read depth), for ChIP-seq and ATAC-seq all sequenced reads were mapped. Peak calling was performed using MACS2 taking a q value of 0.05 as threshold and peaks were filtered for chr2p amplified regions. Homer was used to perform motif enrichment analysis, with 200 bp around the peak summit as input. Overlap of peaks, annotation, heatmaps and pathway enrichment was analysed using DeepTools, the R package ChIPpeakAnno, and the web tool enrichR. Sushiplot was used for visualization of the data upon RPKM normalization or log likelihood ratio calculation with MACS2.

Flow Cytometry

Plots

Confirm that:

- [ ] The axis labels state the marker and fluorochrome used (e.g. CD4-FITC).
- [ ] The axis scales are clearly visible. Include numbers along axes only for bottom left plot of group (a 'group' is an analysis of identical markers).
- [ ] All plots are contour plots with outliers or pseudocolor plots.
- [ ] A numerical value for number of cells or percentage (with statistics) is provided.

Methodology

Sample preparation	*Describe the sample preparation, detailing the biological source of the cells and any tissue processing steps used.*
Instrument	*Identify the instrument used for data collection, specifying make and model number.*
Software	*Describe the software used to collect and analyze the flow cytometry data. For custom code that has been deposited into a community repository, provide accession details.*

Cell population abundance | *Describe the abundance of the relevant cell populations within post-sort fractions, providing details on the purity of the samples and how it was determined.*

Gating strategy | *Describe the gating strategy used for all relevant experiments, specifying the preliminary FSC/SSC gates of the starting cell population, indicating where boundaries between "positive" and "negative" staining cell populations are defined.*

☐ Tick this box to confirm that a figure exemplifying the gating strategy is provided in the Supplementary Information.

Magnetic resonance imaging

Experimental design

Design type | *Indicate task or resting state; event-related or block design.*

Design specifications | *Specify the number of blocks, trials or experimental units per session and/or subject, and specify the length of each trial or block (if trials are blocked) and interval between trials.*

Behavioral performance measures | *State number and/or type of variables recorded (e.g. correct button press, response time) and what statistics were used to establish that the subjects were performing the task as expected (e.g. mean, range, and/or standard deviation across subjects).*

Acquisition

Imaging type(s) | *Specify: functional, structural, diffusion, perfusion.*

Field strength | *Specify in Tesla*

Sequence & imaging parameters | *Specify the pulse sequence type (gradient echo, spin echo, etc.), imaging type (EPI, spiral, etc.), field of view, matrix size, slice thickness, orientation and TE/TR/flip angle.*

Area of acquisition | *State whether a whole brain scan was used OR define the area of acquisition, describing how the region was determined.*

Diffusion MRI ☐ Used ☐ Not used

Preprocessing

Preprocessing software | *Provide detail on software version and revision number and on specific parameters (model/functions, brain extraction, segmentation, smoothing kernel size, etc.).*

Normalization | *If data were normalized/standardized, describe the approach(es): specify linear or non-linear and define image types used for transformation OR indicate that data were not normalized and explain rationale for lack of normalization.*

Normalization template | *Describe the template used for normalization/transformation, specifying subject space or group standardized space (e.g. original Talairach, MNI305, ICBM152) OR indicate that the data were not normalized.*

Noise and artifact removal | *Describe your procedure(s) for artifact and structured noise removal, specifying motion parameters, tissue signals and physiological signals (heart rate, respiration).*

Volume censoring | *Define your software and/or method and criteria for volume censoring, and state the extent of such censoring.*

Statistical modeling & inference

Model type and settings | *Specify type (mass univariate, multivariate, RSA, predictive, etc.) and describe essential details of the model at the first and second levels (e.g. fixed, random or mixed effects; drift or auto-correlation).*

Effect(s) tested | *Define precise effect in terms of the task or stimulus conditions instead of psychological concepts and indicate whether ANOVA or factorial designs were used.*

Specify type of analysis: ☐ Whole brain ☐ ROI-based ☐ Both

Statistic type for inference (See Eklund et al. 2016) | *Specify voxel-wise or cluster-wise and report all relevant parameters for cluster-wise methods.*

Correction | *Describe the type of correction and how it is obtained for multiple comparisons (e.g. FWE, FDR, permutation or Monte Carlo).*

Models & analysis

n/a	Involved in the study
☐	☐ Functional and/or effective connectivity
☐	☐ Graph analysis
☐	☐ Multivariate modeling or predictive analysis

Functional and/or effective connectivity

Report the measures of dependence used and the model details (e.g. Pearson correlation, partial correlation, mutual information).

Graph analysis

Report the dependent variable and connectivity measure, specifying weighted graph or binarized graph, subject- or group-level, and the global and/or node summaries used (e.g. clustering coefficient, efficiency, etc.).

Multivariate modeling and predictive analysis

Specify independent variables, features extraction and dimension reduction, model, training and evaluation metrics.

This checklist template is licensed under a Creative Commons Attribution 4.0 International License, which permits use, sharing, adaptation, distribution and reproduction in any medium or format, as long as you give appropriate credit to the original author(s) and the source, provide a link to the Creative Commons license, and indicate if changes were made. The images or other third party material in this article are included in the article's Creative Commons license, unless indicated otherwise in a credit line to the material. If material is not included in the article's Creative Commons license and your intended use is not permitted by statutory regulation or exceeds the permitted use, you will need to obtain permission directly from the copyright holder. To view a copy of this license, visit http://creativecommons.org/licenses/by/4.0/
